# The Role of Metacognitive Components in Creative Thinking

**DOI:** 10.3389/fpsyg.2019.02404

**Published:** 2019-10-24

**Authors:** Xiaoyu Jia, Weijian Li, Liren Cao

**Affiliations:** ^1^Center for Studies of Education and Psychology of Ethnic Minorities in Southwest China, Southwest University, Chongqing, China; ^2^Institute of Psychology, Zhejiang Normal University, Jinhua, China; ^3^Department of Psychology and Behavioral Sciences, Zhejiang University, Hangzhou, China

**Keywords:** creative thinking, metacognitive knowledge, metacognitive experience, metacognitive monitoring and control, creative process

## Abstract

Metacognition refers to the knowledge and regulation of one’s own cognitive processes, which has been regarded as a critical component of creative thinking. However, the current literature on the association between metacognition and creative thinking remains controversial, and the underlying role of metacognition in the creative process appears to be insufficiently explored and explained. This review focuses on the roles of three aspects of metacognition (i.e., metacognitive knowledge, metacognitive experience, and metacognitive monitoring and control) in creative thinking and offers a primary summary of the neurocognitive mechanisms that support metacognition during creative thinking. Future research is needed to explore the interactive effects of the metacognitive components on creative thinking and to elucidate the function of metacognition during different stages of the creative process.

## Introduction

Metacognition is viewed as the ability to think about one’s current cognitive processes (Flavell, 1976). It is also called “cognition about cognition,” which plays a top-down regulation role in various cognitive processes, such as learning, memory, decision-making, and other high-level cognition (Son and Metcalfe, 2000; Metcalfe, 2002; Ariel et al., 2009). Creativity, a unique ability of human beings, refers to generating original and useful ideas or developing novel solutions to problems under a given context (Runco, 2010; Runco and Acar, 2012; Abraham, 2013). In the past decade, researchers have hypothesized that creative thinking may rely on metacognition components (Davidson and Sternberg, 1998; Berkowitz and Ansari, 2008; Lizarraga and Baquedano, 2013; Erbas and Bas, 2015; Preiss et al., 2016). We believe that a relevant review and discussion of this topic can not only enrich the current theories of creative thinking but also provide a new direction for the cultivation of creativity.

Investigations of the processing mechanism that underlies creative thinking have typically considered metacognition as a single cognitive component, such as self-regulation during representational change and metacognitive self-monitoring or self-confidence when outputting the answer (Hong et al., 2016; Rudolph et al., 2017). Although researchers in the creativity field have emphasized the special role of metacognition, to the best of our knowledge, very few theoretical or empirical studies have clarified how metacognition affects creative thinking. Early on, researchers emphasized creative thinking as a self-regulated metacognitive process (Pesut, 1990). For example, some researchers have advanced the concept of “creative metacognition,” which is a combination of self-knowledge (e.g., knowing one’s own creative advantages and disadvantages in a certain field) and contextual knowledge (e.g., knowing when/where/how/why to be creative, Feldhusen and Goh, 1995; Davidson and Sternberg, 1998; Kaufman and Beghetto, 2013). In addition, a few empirical studies have examined the relationship between metacognition and creative thinking in terms of the following three aspects: (1) exploring the positive/negative correlation between metacognition and creative thinking *via* behavioral investigation (Lizarraga and Baquedano, 2013; Erbas and Bas, 2015; Hong et al., 2016; Preiss et al., 2016); (2) understanding the function of brain regions activated in creative thinking from the metacognition perspective, for example, the anterior cingulate gyrus (ACC) and dorsolateral prefrontal cortex (DLPFC, Geake and Hansen, 2005; Berkowitz and Ansari, 2008; Kounios et al., 2008); and (3) enhancing individual creative thinking by metacognitive training (Hargrove, 2013; Abdivarmazan et al., 2014; Hargrove and Nietfeld, 2015). Although theoretical and empirical studies have indicated that metacognition may be critically involved in creative thinking, the conclusion regarding whether metacognition has a positive or negative effect on creative thinking and how it engages in the creative process remains controversial. Therefore, this article systematically disentangles the roles of the three components of metacognition in creative thinking and discusses several central issues in the current literature to guide future research.

## The Construct of Metacognition

In general, metacognition refers to individuals’ ability to have knowledge, awareness, and control of their cognitive activities (Nelson, 1990). The concept of metacognition is regarded as being fuzzy with indistinct boundaries, as researchers have often classified it into the three interconnected components of metacognitive knowledge, metacognitive experience, and metacognitive monitoring and control (Flavell, 1979). Specifically, metacognitive knowledge, which refers to the declarative knowledge of cognitive processes and products (Dowson and Mcinerney, 2004; Efklides, 2011), has generally been divided into personal knowledge (e.g., hobbies, memory characteristics, ways of thinking, and ability limitations); task knowledge (e.g., task structures, task goals); and strategic knowledge (e.g., advantages or disadvantages and the applicability of each strategy). Metacognitive experience, the cognitive or emotional experience that accompanies cognitive activity, can occur in the early, middle, and late stages of cognitive activity (Flavell, 1979). Metacognitive experience is not a cognitive operation itself but an individual’s subjective perception of the ease or difficulty of certain cognitive operations (Rummer et al., 2016). In addition, metacognitive monitoring and control refers to an individual’s self-conscious supervision and regulation of the cognitive processes. Specifically, metacognitive monitoring includes individuals ability to plan, monitor, and evaluate their cognitive activities, followed by subsequent metacognitive control that allows individuals to regulate their cognitive processes, such as adjusting task goals, distributing study time, and selecting cognitive strategies (Flavell, 1979).

## The Construct of Creative Thinking

A standard definition of creativity has lacked consensus, as the construct of creativity is complex and different disciplines have distinct focuses. Early researchers were more likely to consider creativity as a personal trait, such as personality (Guilford, 1950; Eysenck, 1993). With the development of experimental technology in the field of psychology, especially neuroimaging methods, a clear operational definition may benefit from investigations into the nature of creativity; thus, most researchers have viewed creativity as a problem-solving ability, namely, the ability to imagine novel or useful ideas or products in a given context (Sternberg and Lubart, 1999; Runco, 2010). In addition, some comprehensive frameworks have attempted to describe a profile of creativity. Batey (2012) proposed an integrative perspective of the 4-P model of creativity by emphasizing the following four dominant factors of creativity: person—individual traits or characteristics; process—thought process involved in the creation of ideas; press—environmental influences; and product—output from creative activity. These four factors are highly interrelated, as a product is created by a series of cognitive processes that a person uses in a specific environment. Furthermore, it can be recognized that the lack of a consensual definition of creativity has led to a multitude of measurement approaches. A review of research methods in creativity studies (2003–2012) revealed that researchers have relied heavily on divergent thinking tests, problem-solving tasks or products to assess creativity (Long, 2014).

Eysenck (1993) framed divergent-convergent interactions as important to conceptualizations of creativity. That is, creativity could be described as a constant oscillation between divergent and convergent thinking (Finke et al., 1992; Bink and Marsh, 2000). Specifically, divergent thinking refers to the expansive generation of novel ideas for an open-ended problem, whereas convergent thinking emphasizes producing a single response from all possible answers to a given problem (Guilford, 1967). The differences between these two types of creative thinking lead to distinct measurement approaches. Generally, divergent thinking can be assessed by a diverse set of tasks, such as the classic Alternative Uses Task (AUT, Guilford, 1967), Torrance Test of Creative Thinking (Wallach and Torrance, 1968), and Multiple Choice Test (Auzmendi et al., 1996). The degree of divergent thinking (i.e., scoring) mainly depends on the sum of fluency, flexibility, and originality of ideas. In contrast, convergent thinking is typically assessed by the Remote Associations Test (RAT, Mednick, 1962), insight problem-solving tasks (Luo and Knoblich, 2007), and creative analogical reasoning (Zhang et al., 2014). Although these two prominent measures do not guarantee actual creative thinking performance, compelling evidence well supports the construct validity of the two psychometric tasks for creative thinking.

## The Intersection of Metacognition and Creative Thinking

Creative thinking can be regarded as a metacognitive process in which the combination of individual’s cognitive knowledge and action evaluation results in creation. Specifically, creative thinking involves a series of cognitive processes, such as the acquisition of knowledge and skills, the transformation of knowledge into new forms, and the verification of products from internal and external standards (Amabile, 1983). It seems to be appropriate to involve metacognition in these stages due to its crucial role in high-level cognition. For example, for any creative action to be successful, relevant prior knowledge must be consciously selected, and a work plan must be implemented. Moreover, the strategies must be flexibly adjusted, and the originality and utility of products must be evaluated. In fact, all of these functions are metacognitive in nature, and their use would likely enhance creativity (Armbruster, 1989). Accordingly, we systemically review the role of the three components of metacognition in creative thinking.

## Metacognitive Knowledge and Creative Thinking

Metacognitive knowledge guides individuals to select, evaluate, and correct cognitive strategies, which are important for creative thinking. Empirically, several works have shown that individual’s metacognitive knowledge contributes to domain-specific creativity. For example, Lizarraga and Baquedano (2013) found a moderate correlation between metacognitive knowledge and visual-spatial creativity (e.g., drawing and titling four drawings from provided lines), and similar findings were reported on mathematic creativity (Erbas and Bas, 2015). Fayenatawil et al. (2011) adopted a protocol analysis to examine both artists and non-artists during the creation of original drawings. The results revealed that artists who possess much more metacognitive knowledge of plans, goals, and descriptions performed better than non-artists in an artistic creation task. Additionally, Zeng et al. (2011) constructed a conceptual model of the IT creativity of studying and designing computer hardware or software and found that the metacognitive knowledge about explicit problem analysis, remote association, abstraction, and domain-specific knowledge played important roles in the analysis, ideation, evaluation, and implementation of IT creativity, respectively.

Several intervention studies have found that the training of metacognitive knowledge promotes creative problem solving. For instance, Abdivarmazan et al. (2014) used a pretest-posttest design to examine the effect of training metacognitive knowledge for problem solving. The subjects were divided into an experimental group and a control group. The experimental group received metacognitive strategy knowledge training a total of eight times (50 min each time), while the control group did not receive any intervention. The results showed that metacognitive knowledge training can significantly improve creative problem solving. This intervention effect is consistent with previous findings (Hargrove, 2013).

Nevertheless, Preiss et al. (2016) found no correlation between individual metacognitive knowledge and creative thinking. In their study, the AUT and the compound word association task were used to measure creative thinking, and the self-reporting scale was used to evaluate individual declarative strategic knowledge about planning, monitoring, and regulating (Dowson and Mcinerney, 2004). The results showed that metacognitive knowledge did not significantly predict the performance in either of the two creative thinking tasks after controlling for fluid intelligence and reading difficulties.

Not all empirical studies have found a positive correlation between metacognitive knowledge and creative thinking, and several limitations should be considered. First, an individual’s metacognition knowledge assessed through a self-report approach (Antonietti et al., 2000; Hargrove and Nietfeld, 2015; Preiss et al., 2016) has been debated due to potential problems with its reliability and validity. Previous studies have suggested that unskilled individuals always exaggerate their self-assessment because they have poor analytical ability (Kruger and Dunning, 1999) and are overly interested in motivations and intentions (Kruger and Gilovich, 2004; Pronin, 2008). Similarly, Preiss et al. (2016) suggested that the self-report method may not accurately reflect metacognitive knowledge—especially the metacognitive strategic knowledge of planning, monitoring, and regulation—for individuals who have difficulty in recognizing their abilities. Second, there is a dissociation between self-report metacognitive knowledge and its application to specific tasks, and self-report metacognitive knowledge may not directly affect task performance (Scherer and Tiemann, 2012; Hargrove and Nietfeld, 2015). Third, existing studies have mainly focused on the role of metacognitive strategic knowledge in creative thinking, whereas an examination of the other two variables (personal knowledge and task knowledge) is disregarded. For example, creative mindsets, a type of metacognitive knowledge that refers to individuals’ incremental or entity-mindset view of creativity (e.g., Creative mindsets, O’Connor et al., 2013), may influence their creative performance (O’Connor et al., 2013; Karwowski, 2014). That is, individuals with different types of creativity mindsets have different cognitive processing characteristics, such as having different ways of learning, orienting toward a target, making strategic choices, and cognitive persistence (Dweck and Leggett, 1988; De Dreu et al., 2008; Baas, 2010; Benedek et al., 2011; Roskes et al., 2012), which are regarded as critical aspects of creativity. It is inferred that creative mindsets may indirectly influence creativity through other cognitive variables. Therefore, more empirical studies are needed to uncover the mechanism of the different components of metacognitive knowledge in creative thinking.

## Metacognitive Experience and Creative Thinking

Numerous empirical studies have confirmed that metacognitive experience can be indicated by the metacognitive cue of processing fluency (Koriat et al., 2004; Oppenheimer, 2008; Alter and Oppenheimer, 2009b; Jia et al., 2016). Processing fluency, the subjective feeling of the ease of information processing (Koriat et al., 2004), influences a variety of cognitive tasks, such as factual preferences, aesthetic appreciation, brand assessment, and reading comprehension (Alter and Oppenheimer, 2009a; Miele and Molden, 2010). For the relationship between processing fluency and creative thinking, previous research has revealed that processing fluency affects a series of cognitive activities involved in creative thinking (Gilhooly et al., 2007) such as goal setting (Storbeck and Clore, 2007), work efforts (Miele and Molden, 2010), strategy choice (Lucas and Nordgren, 2015), and processing styles (Alter et al., 2007).

Mehta et al. (2012) asked 95 participants to complete the AUT and RAT with different levels of background noise. Meanwhile, a 7-point scale that contained three questions was used to assess the subjective level of processing disfluency. The results showed that a moderate (vs. low) level of noise induced higher processing disfluency and consequently enhanced creative thinking performance. Alter and Oppenheimer (2009a) suggested that processing disfluency could induce individual’s higher construal thinking and less attention-focused, which were beneficial to creative thinking.

Moreover, processing fluency could also influence creative thinking by inducing different types of processing styles. Alter et al. (2007) argued that processing fluency can induce different degrees of intuition and analytical processing. That is, if information processing is perceived as easy and fluent, much more intuitive processing will be activated; conversely, if information processing is perceived as difficult and disfluent, a much greater analytical processing style will be activated (Kuhl et al., 2014). Mehta et al. (2012) found that the disfluent processing experience allows individuals to use more analytical processing, which, in turn, promotes creative thinking performance as measured by both the AUT and RAT. In addition, neurophysiological evidence has revealed that processing disfluency induces the activation of the anterior cingulate cortex (Boksman et al., 2005) and the prefrontal cortex (PFC), which allows people to think thoughtfully and use analytical processing to complete creative tasks (Goel et al., 2000; Botvinick et al., 2001; Lieberman et al., 2002). Taken together, these results suggest that processing disfluency could promote creative thinking by activating a much higher level of analytical processing.

Nevertheless, the notion that overly analytical processing induced by processing disfluency impedes convergent thinking has been supported by some studies (Friedman and Forster, 2005; Aiello et al., 2012). For example, in the study by Aiello et al. (2012) in which both bilingual and monolingual participants completed the RAT before or after an artificial grammar task with or without the “use your gut” instruction (just go with your “gut feeling” to make a decision), the results showed that the completion of an artificial grammar task with the “use your gut” instruction before enhanced the RAT performance, suggesting the beneficial role of a less analytic approach in the RAT performance. Similarly, another effective indicator of convergent thinking—insight problem solving, which involves an “aha!” experience that the solution could occur in a sudden and unpredictable manner with little or no conscious processing, has been confirmed to be inhibited much more by analytical processing (Metcalfe and Wiebe, 1987; Qiu and Zhang, 2008).

Whether the metacognitive experience reflected by processing fluency promotes or inhibits creative thinking is controversial. There are several reasons for this controversy. First, different types of creative thinking, such as divergent and convergent thinking, may have different relationships with processing fluency. According to Benedek et al. (2011), different types of creative thinking have significant differences in processing mechanisms. Specifically, divergent thinking tasks involve analytical processing (Unsworth et al., 2011), whereas too much analytical processing may inhibit convergent thinking tasks as a requirement of a novel representation for problems and the search for remote connections to memory (Metcalfe and Wiebe, 1987). Therefore, the differentiated roles of processing fluency in divergent and convergent thinking should be considered. Second, the problem of the classification and operation of the metacognitive experience may be partly responsible for the controversial results. Previous studies, however, have paid less attention to exploring this issue. To be more specific, processing fluency, an indicator of the metacognitive experience which has always been used in previous studies, could be divided into perceptual fluency, encoding fluency, and retrieval fluency, whereas these distinct types of processing fluency may have different effects on different types of creative thinking (Koriat et al., 2004). For example, the AUT, which requires individuals to generate as many novel ideas as possible, was relied on the fast and effective strategic memory retrieval ability (Forthmann et al., 2019). That is, the retrieval fluency could play a key role in the creative ideas production. Conversely, the RAT requires individuals to generate a target word from a set of cue words, which means that perceptual and encoding fluency may influence the results. Third, the indirect ways of manipulating processing fluency, such as pre-experiment tasks or noise activation, are greatly affected by additional factors beyond the experiment (Mehta et al., 2012). Therefore, the direct ways of disrupting subjective feelings of fluency, such as font style manipulation (Alter and Oppenheimer, 2009b; Jia et al., 2016), semantic priming (Winkielman and Cacioppo, 2001), and statement-background color contrast (Hansen et al., 2008), should be investigated in future studies.

## Metacognitive Monitoring and Control and Creative Thinking

It is worth mentioning that metacognition can be divided into the “knowledge of cognition” and the “regulation of cognition” by using a dichotomy (Brown, 1978). The regulation of the cognition component includes individual’s planning, examining, monitoring, testing, and evaluating cognitive activities, which corresponds to “metacognitive monitoring and control.” Thus, we now comprehensively introduce the relationship between “metacognitive monitoring and control” and the “regulation of cognition” and creativity.

Sternberg (1985) argued that the process of creative thinking involved “self-monitoring” by monitoring other components through metacognition. Evidence from cognitive neuroscience studies reveals that the brain regions responsible for creative thinking overlap with the activated brain regions in metacognition monitoring and control, which mainly involve the dorsolateral prefrontal and ventrolateral prefrontal cortexes (Carlsson et al., 2000; Zysset et al., 2001). Empirically, Zhang and Xiao (1996) asked participants to complete the Mutilated Chickboard problem, which requires people to change the representation from the space of all possible coverings to the “meta-level” space to find the correct problem representation. Their results showed that successful problem solvers were better at monitoring, transforming, and adjusting their search strategies according to changeable problem conditions, suggesting the positive effect of metacognitive monitoring and control on creative problem solving. Similarly, Xing and Chen (2009) further revealed that individuals with higher metacognitive monitoring and control abilities showed better performance at solving a Chinese logogriph task (i.e., a type of creative problem-solving task in which participants respond to puzzles) than individuals with a lower ability. The process monitoring theory proposed by Macgregor et al. (2001) explains that metacognitive monitoring and control ability can constantly monitor the gap between the existing state and the target state and then adjust cognitive strategies to access creative problem solving.

Moreover, intervention studies have shown that metacognitive skills training could promote creative thinking (Atman et al., 2005). For example, Hargrove (2013) divided participants into an intervention group and a control group by counterbalancing their professional categories and genders. The participants in the intervention group received 1–2 semesters (17 h/semester) of metacognitive skills training to learn how to plan and implement thinking strategies, how to monitor and evaluate the quality of thinking, and how to amend incorrect thinking, whereas the participants in the control group received only professional courses every semester. The participants in the intervention group showed a significantly higher level of creative thinking as measured by the RAT and an art design task. A similar effect was also reported by Hargrove and Nietfeld (2015).

Since Kaufman and Beghetto (2013) proposed the concept of creative metacognition, a growing number of studies have attempted to examine creative metacognitive monitoring accuracy, which can be assessed by comparing a general self-external assessment. Silvia et al. (2008) required participants to complete the AUT and then asked them and external raters to indicate the most creative responses from the reaction pool. The results showed that when the level of creative ability was higher, the participants more accurately monitored their responses. Beghetto et al. (2011) further examined this issue by asking primary school students to assess their creative ability in mathematics and science and observed that creative metacognitive monitoring accuracy (one type of metacognitive knowledge) can significantly explain the instructor’s assessment of creative ability. Priest (2006) and Kaufman et al. (2010), however, did not find significant correlations between creative thinking and metacognitive monitoring and control in the art, writing, and musical fields.

The lack of a correlation between creative thinking and metacognitive monitoring and control can be found in other empirical studies. Metcalfe (1986) asked participants to make feeling-of-knowing judgments, an index of metacognitive monitoring (Maclaverty and Hertzog, 2009), for creative problems and then give corresponding answers within 5 min. If the participants realized that the correct answer was closer, the value of the feeling-of-judgment would be higher. However, the results showed that the value of the feeling-of-judgment did not relate to the probability of producing the correct answer. Hong et al. (2016) asked participants to complete a divergent thinking task of creating a new cultural environment and to answer eight questions, such as “I always monitor my job completion process,” in order to measure their metacognitive plans and monitoring. The results showed that individuals’ metacognitive monitoring had no significant effect on their divergent thinking performance.

Overall, the conclusion that a positive correlation exists between metacognitive monitoring and control and creative thinking may not be as stable as we expected. In fact, metacognitive monitoring and control includes a set of subcomponents, such as goal setting, planning execution, strategy selection, and cognitive assessment (Flavell, 1976). Many previous studies have either focused on either one or some subcomponents of metacognitive monitoring and control. For example, Hong et al. (2016) explored only the effects of planning and monitoring subcomponents on divergent thinking but summarized the results at the overall level. This method is likely to result in biased or conflicting evidence. In addition, according to Kelemen et al. (2000), there are both “trait” and “situational” metacognitive monitoring and control, which have different concepts and measurements (Veenman et al., 2004; Preiss et al., 2016). Hong et al. (2016) found that individuals’ situational metacognitive monitoring and control have no significant effect on creative thinking while controlling for the variable of an individual’s trait metacognitive monitoring and control. Therefore, their confusion in related studies could at least partly account for the inconsistency in the related results. Generally, all of the above problems should be considered to obtain a better understanding of the relationship between creative thinking and metacognitive monitoring and control.

## Neurophysiological Evidence of Metacognition and Creative Thinking

The general framework of metacognition is characterized by the interplay of meta-level and object-level information (Nelson, 1990). The object-level refers to one’s current cognitive processes (e.g., perception, attention, and decision making), which are monitored or controlled at the meta-level. Previous cognitive neuroscience evidence suggests that the PFC plays a central role in the processing of meta-level top-to-bottom adjustment of the object-level (Fernandezduque et al., 2000). Specifically, the PFC regulates the posterior cortical circuit involvement in object-level processing through a filtering mechanism. In recent years, there has been increasing interest in identifying the regions in the PFC involved in metacognition, including the lateral prefrontal cortex (LPFC), medial prefrontal cortex (mPFC), and DLPFC. These brain regions are responsible for different functions in metacognition (Christoff et al., 2003; Fleming et al., 2010; Fleming and Dolan, 2012). For example, a metacognitive assessment of cognitive tasks (e.g., working memory, episodic memory retrieval, and abstract thinking) induces greater activation of the lateral PFC (Braver and Bongiolatti, 2002; Christoff et al., 2009), whereas metacognitive judgment generally activates the rostral medial prefrontal cortex (RMPFC, prospective judgment), the rostral lateral prefrontal cortex (RLPFC, retrospective judgment, Fleming and Dolan, 2012) and the DLPFC. An fMRI study found that the DLPFC and VLPFC were activated when tasks involved the metacognitive inhibition of sensory information, whereas the DMPFC and DLPFC were activated when tasks (e.g., the Stroop task) involved metacognitive control for concurrent conflicts (Zysset et al., 2001).

Recently, some noninvasive brain stimulation and lesion studies have suggested that a disabled PFC can affect metacognitive monitoring in perceptual decision making (Cul et al., 2009; Rounis et al., 2010; Ham et al., 2014). Despite this, the neural mechanism that underlies individual metacognition remains controversial. One core component of this controversy is whether functional segregation exists in the prefrontal system that is specific to metacognition. Qiu et al. (2017) used a novel decision-redecision paradigm, in which participants make an initial decision on perceptual and rule-based decision-making tasks (decision phase) followed by another decision on the same tasks (redecision phase), to examine the underlying neural substrates of metacognition on decision making. The results revealed that the dACC is responsible for decision uncertainty monitoring, while the FPC is responsible for the metacognitive control of decision adjustment, suggesting a disconnected role in the PFC and a distinct role in metacognition.

Interestingly, these studies of metacognition show brain recruitment (e.g., ACC, IFG, mPFC, and DLPFC) similar to that in creative thinking (Dietrich and Kanso, 2010; Fink et al., 2012; Fox and Christoff, 2014). Specifically, the LPFC (including the IFG and DLPFC) is essential to various creativity (Aziz-Zadeh et al., 2010). Similarly, the ACC, which plays a role in the solutions monitoring, was also confirmed to be activated during the creative process (Geake and Hansen, 2005; Berkowitz and Ansari, 2008; Kounios et al., 2008). A study regarding musical improvisation found that pianists exhibited stronger activation in the ACC under conditions of rhythmic and melodic freedom, suggesting the positive effect of metacognition for monitoring the conflicts among different melodies or rhythms on the creative process (Berkowitz and Ansari, 2008).

In addition, several fMRI studies have reported that metacognition is associated with the anterior insula, which is responsible for promoting the individual consciousness of emotional and physical states (Craig, 2009), and for delivering this information to PFC areas (Fleming and Dolan, 2012). For example, people who have had mindfulness training are more likely to perceive their thoughts, emotions, and physical state; in addition, they show stronger activation in the insula and the lateral prefrontal cortex (McCaig et al., 2011). Similarly, the mPFC and the anterior insula can also be activated in the generation stage in multiple creative tasks (Geake and Hansen, 2005; Howard-Jones et al., 2005; Limb and Braun, 2008). According to the two-stage theory of the creative process (Ellamil et al., 2016), metacognition might play different roles in idea generation and idea evaluation. A low level of metacognitive control can make more diverse pieces of information appear to enter the mind to construct more novel ideas at the stage of idea generation, whereas at the stage of idea evaluation, the activation of the metacognitive system can contribute to evaluations of the novelty and utility of the spontaneous ideas generated during the previous stage. Based on this framework, the latter process may possibly be associated with cognitive activation and positive emotion, which further guides the ensuing idea generation. This neurophysiological evidence from the aforementioned studies reveals that the prefrontal regions related to metacognition are involved not only in monitoring but also in value evaluation and emotion in the creative process.

## Concluding Remarks and Future Directions

The present study primarily focuses on the intersection between metacognition and creative thinking. Although increasing research points out that metacognition may play an important role in creative thinking, the empirical studies reviewed in the present study have not reached a consensus. Some obvious limitations remain. First, previous studies have mainly applied correlational approaches to investigate the intersection between metacognition and creative thinking and have neglected to reveal the cause-and-effect between the two constructs. Future research is particularly essential to explore the internal mechanism of metacognition that affects creative thinking. Second, the reliability and validity of metacognition measurements are controversial. Specifically, self-reporting is greatly influenced by subjective expectations, whereas a think-aloud protocol is time consuming, and discourse analysis is subject to the quality of the interpersonal interaction among groups (Desoete, 2008). To avoid unexpected factors generated by these methods, objective measurement indexes such as prospective monitoring, retrospective monitoring, and the judgment of confidence (Bjork et al., 2013) could be promising ways to assess metacognition. Third, the three components of metacognition are independent but closely interrelated (Dowson and Mcinerney, 2004; Efklides, 2011). Previous research has always focused on metacognition as a whole or a single component, which has led to the lack of an interaction effect of the three metacognitive subcomponents on creativity. Fourth, the differentiated effects of metacognition on different types of creative thinking have not yet been described. Accordingly, we discuss two important directions in future research as follows.

### Exploring the Role of Metacognition in the Creative Process

According to the aforementioned 4-P model of creativity (Batey, 2012), it should be acknowledged that most of the previous studies have tended to explore the relationship between metacognition and creativity outcomes (e.g., responses in the AUT) but have neglected to discuss the role of metacognition during the dynamic creative process. The creative process, namely, the sequence of thoughts and actions that leads to novel, adaptive productions (Lubart, 2001), has been identified as the combination of a series of cognitive processes. According to the classic four-stage model proposed by Guilford (1950), the creative process can be divided into the following four stages: reparation—consciously define and establish the problem; incubation—no conscious mental work on the problem; illumination—the promising idea breaks through to conscious awareness; and verification—evaluate and refine ideas. Whether metacognition plays a different role in different stages of the creative process remains an open question. Armbruster (1989) suggested that the role of metacognition in incubation may be unconscious, whereas it is conscious in verification. Similarly, the geneplore model of the creative process (i.e., idea generation, namely, operating on unstructured, illogical thoughts to produce ideational materials, and idea evaluation, namely, controlling, evaluating, and selecting the best ideas) suggests that the idea generation stage requires no participation of metacognition to produce many more ideas, whereas the idea evaluation stage needs the participation of metacognition to assess the originality and usefulness of ideas (Fox and Christoff, 2014). In addition, Shen et al. (2013) demonstrated that P2 in processing creative problems, as a stimulus-driven frontal metacognitive mechanism, reflects preconscious awareness of the mental impasse at a relatively early rather than the late stage of creative problem solving.

Nevertheless, in investigations of the current issue, regarding metacognition as a whole remains controversial due to its complex construct. Perhaps different components of metacognition have different effects on the creative process. In a recent study, for example, Jankowska et al. (2018) integrated the psychometric approach, eye-tracking methodology, and thinking-aloud protocols and found that the three categories of metacognition play different roles in the creative process. Specifically, one category of exploratory activities was demonstrated to be essential in the initial phase of the creative process, while another two categories, decision-making and control activities and affective-evaluation activities, were involved in the entire creative process. From this independent point of view, we propose that the effect of metacognitive monitoring can be separated from metacognitive control on the creative process. According to the monitoring-affect-control hypothesis (Nelson and Leonesio, 1988), metacognitive control may be the result of prior metacognitive monitoring (Metcalfe and Finn, 2008). For instance, individuals can adjust the strategy selection (an indicator of metacognitive control, Beaty and Silvia, 2012) during the generation of the next idea according to self-assessment of previous ideas originality (an indicator of metacognitive monitoring, Silvia et al., 2008). In this case, we believe that separating the two subcomponents leads to a better understanding of the dynamic monitoring-affect-control process in creative thinking.

Although the three key components of metacognition have been discussed separately, these components are not independent as an interactive system (Efklides, 2011). That is, metacognitive monitoring and control could be activated by relying on metacognitive knowledge and the information provided by metacognitive experiences about the flow of cognitive processing. Accordingly, how these three factors interact in the process of creative thinking remains unclear. Here, we take a creative metacognitive monitoring accuracy, processing fluency (an index of metacognitive experience), and metacognitive monitoring accuracy as examples. Previous studies have demonstrated that individuals with different types of creative mindsets exhibited significant differences in their interpretation of the experience of process disfluency (Miele et al., 2011) and in their level of metacognitive monitoring accuracy (Blackwell et al., 2007). When completing a creative thinking task, individuals with an incremental creative mindset could interpret their processing disfluency as lacking in effort and would show much greater cognitive persistence, whereas individuals with an entity creative mindset could interpret it as an ability deficiency and would give up on further cognitive persistence. Meanwhile, individuals with an incremental creative mindset showed better performance in the metacognitive monitoring of the selecting and evaluating strategies than individuals with an entity creative mindset.

Under the framework of the dynamical creative process, the effect of metacognition components and their interaction on the creative process could be helpful for understanding the current work. Future research could examine the independence and interaction effect of the metacognitive components on the creative process using multiple methods.

### Cultivating Creativity From the Perspective of Metacognition

The practical implication that should be considered is how to foster individual creative thinking from the perspective of metacognition. Apart from teaching individuals’ metacognitive skills (Scott et al., 2004; Hargrove, 2013; Hargrove and Nietfeld, 2015), a new training perspective based on metacognition knowledge could prove to be a novel avenue for creativity cultivation in future studies. This promising example of a metacognition training is creative mindset intervention. A creative mindset, metacognitive knowledge that refers to individuals’ domain-specific implicit theories of creativity aforementioned, could have independent and interactive effects on creativity (Blackwell et al., 2007; Miele et al., 2011). The main idea is that an incremental creative mindset (viewing creativity as malleable and changeable) is beneficial to creativity compared with an entity creative mindset (viewing creativity as stable and unchangeable). More importantly, similar to the idea that it is possible to successfully intervene in a general mindset (Hong et al., 1999; Blackwell et al., 2007; Paunesku et al., 2015), it is possible to intervene in a creative mindset.

A general mindset intervention has been a popular topic in many disciplines such as learning, writing, anxiety, and musicality (Donohoe et al., 2012; Müllensiefen et al., 2015; Paunesku et al., 2015; Schleider and Weisz, 2018), and improving creativity through creative mindset intervention shows promise. The general mindset intervention methods that aim to encourage an incremental mindset could be transferred to and borrowed by the creativity field. For example, Hong et al. (1999) asked students to read articles in popular magazines to emphasize the importance of environmental factors rather than genetic components to mindset development. Blackwell et al. (2007) succeeded in altering the mindsets of middle school students over the course of eight intensive sessions that focused on the study strategies of brain plasticity and ways that their mindset changes over time. Other researchers have also used a similar design in their intervention methods (Aronson et al., 2002; Yeager et al., 2013). Bostwick (2015) summarized that the key commonalities of these intervention designs included three factors, namely, the “saying is believing aspect” (the article was read), “students formalized it in their own words” (the article was understood), and the “interventional time point of students’ most susceptible to the intervention.” Future research could attempt to create a series of standardized creative mindset interventions to contribute to creativity cultivation.

## Author Contributions

All authors listed have made a substantial, direct and intellectual contribution to the work, and approved it for publication.

### Conflict of Interest

The authors declare that the research was conducted in the absence of any commercial or financial relationships that could be construed as a potential conflict of interest.
